# Metallic Flexible NiTi Wire Microcrack Transducer for Label-Free Impedimetric Sensing of *Escherichia coli*

**DOI:** 10.3390/bios16010054

**Published:** 2026-01-10

**Authors:** Gizem Özlü Türk, Mehmet Çağrı Soylu

**Affiliations:** 1 Biological and Medical Diagnostic Sensors Laboratory (BioMeD Sensors Lab.), Department of Biomedical Engineering, Faculty of Engineering, Erciyes University, Kayseri 38030, Türkiye; gizemozluturk@yyu.edu.tr; 2 Biomedical Engineering Program, Graduate School of Natural and Applied Sciences, Erciyes University, Kayseri 38280, Türkiye; 3 Biomedical Device Technologies Program, Department of Electronics and Automation, Ercis Vocational School, Van Yuzuncu Yil University, Van 65400, Türkiye

**Keywords:** flexible nickel–titanium (NiTi) biosensor, label-free impedimetric sensing, martensitic microcracks, point-of-care biosensing, self-healing interfaces

## Abstract

Flexible biosensors offer rapid and low-cost diagnostics but are often limited by the mechanical and electrochemical instability of polymer-based designs in biological media. Here, we introduce a metallic flexible microcrack transducer that exploits the intrinsic deformability of superelastic nickel–titanium (NiTi) for label-free impedimetric detection. Mechanical bending of NiTi wires spontaneously generates martensitic-phase microcracks whose metal–gap–metal geometry forms the active transduction sites, where functional interfacial layers and captured analytes modulate the local dielectric environment and govern the impedance response. Our approach imparts a novel dielectric character to the alloy, enabling its unexplored application in the megahertz (MHz) frequency domain (0.01–10 MHz) where native NiTi is merely conductive. Functionalization with *Escherichia coli (E. coli)*-specific antibodies renders these microdomains biologically active. This effectively transforms the mechanically induced microcracks into tunable impedance elements driven by analyte binding. The γ-bent NiTi sensors achieved stable and quantitative detection of *E. coli* ATCC 25922 in sterile human urine, with a detection limit of 64 colony forming units (CFU) mL^−1^ within 45 min, without redox mediators, external labels, or amplification steps. This work pioneers the use of martensitic microcrack networks, mimicking self-healing behavior in a superelastic alloy as functional transduction elements, defining a new class of metallic flexible biosensors that integrate mechanical robustness, analytical reliability, and scalability for point-of-care biosensing.

## 1. Introduction

Flexible biosensors are critical to rapid, low-cost, and point-of-care diagnostics, enabling real-time biochemical monitoring in wearable and disposable platforms [1,2]. Among various designs, polymer-based substrates such as hydrogels, elastomers, and conductive composites have dominated recent developments owing to their softness and adaptability to biological interfaces [3,4]. However, the long-term performance of these soft matrices is often limited by mechanical fatigue, swelling or dehydration, and chemical aging under repeated operation, which can lead to signal drift and poor reproducibility. These limitations motivate the development of flexible transducers that combine conformal contact with improved structural and chemical robustness beyond conventional polymer architectures [5].

To address these challenges, several approaches have integrated metallic nanostructures or conductive films onto soft polymer backings, allowing the substrate to absorb mechanical strain while the metallic layer provides electrochemical activity [2,6]. At the same time, the flexibility of such systems still depends on the integrity of the polymer support, and repeated bending or stretching can cause cracking, delamination, or loss of adhesion in the conductive layer [7]. An alternative strategy is to employ intrinsically flexible metals whose mechanical compliance originates from solid-state phase transformations rather than external soft supports.

Nickel–titanium (NiTi) shape-memory alloys combine high fatigue resistance, corrosion resistance, and good biocompatibility and are widely used in devices such as stents, filters, and orthodontic wires [8,9]. Their superelastic behavior is governed by stress-induced martensitic transformation and accommodates several percent of reversible strain without permanent plastic deformation, during which the alloy naturally forms microcracks that mimic self-healing behavior by opening under stress and closing upon unloading [10]. NiTi has also been used directly as an electrochemically active substrate, for example, by forming Ni–Ti–O or Ni(OH)_2_/TiO_2_ oxide layers in situ for non-enzymatic glucose sensing, showing that mechanically robust NiTi wires can support stable redox-active interfaces [11,12]. Within the wider scope of MEMS, however, NiTi applications have been predominantly restricted to mechanical functions, ranging from low-frequency thermal actuators [13] to high-frequency mechanical resonators [14]. Unlike these conventional approaches that rely on resistive heating, the high-frequency dielectric potential of the alloy has remained unexplored. Consequently, the capacity of NiTi to operate as a pseudocapacitive transducer in the megahertz regime (0.01–10 MHz) is unknown. Here, we hypothesize that the formation of confined, bio-functionalized microcrack cavities introduces a new dielectric mechanism that unlocks this high-frequency capability.

Urinary tract infections (UTIs) are among the most common bacterial infections worldwide, and Escherichia coli (*E. coli*) is responsible for the majority of uncomplicated cases and was selected as a model pathogen and clinically relevant target for rapid screening assays. In routine clinical microbiology, significant bacteriuria is traditionally defined as ≥10^5^ CFU mL^−1^ in midstream urine, although lower diagnostic thresholds (10^3^–10^4^ CFU mL^−1^) are accepted in acute cystitis in women and catheter-associated infections [15]. Culture-based identification typically requires 18–48 h, and although various optical, electrochemical, and lateral-flow assays have been proposed, many still rely on labels, redox mediators, or multi-step liquid handling, underscoring the need for simple label-free sensors capable of detecting E. coli directly in urine with clinically relevant limits of detection [16].

**Figure 1:**
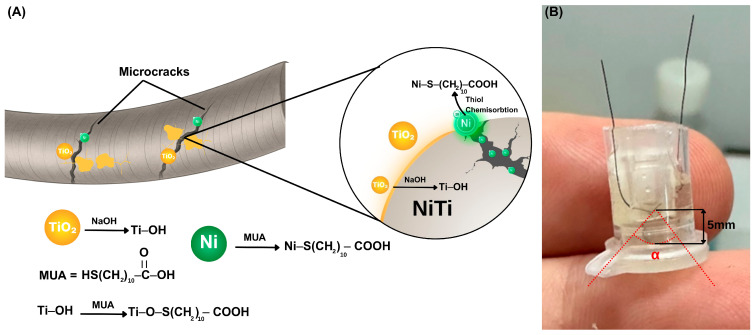
(**A**) Schematic illustration of the surface functionalization steps, showing hydroxylation and MUA self-assembly on both the native surface and microcrack interiors. (**B**) Photograph of the practical single-wire sensor configuration. The γ-bent segment (α = 55°, bending diameter ≈ 5 mm) defines the active sensing region immersed in solution, while the non-exposed ends of the same continuous NiTi wire serve as electrical contacts.

In this study, we convert a superelastic NiTi wire into an active impedimetric biosensor by exploiting its intrinsic phase transformation properties (Figure 1). Bending the wire into a γ-shaped configuration triggers a stress-driven martensitic transformation, which creates a network of reversible microcracks. Here, we strategically repurpose these stress-induced defects—conventionally viewed as structural failures—to serve as confined, nickel-rich transduction cavities. Simultaneously, the exterior of the wire retains its native TiO_2_ passivation layer. Upon NaOH activation, both the nickel-rich microcrack interiors and the TiO_2_-covered outer regions participate in thiol-based surface functionalization using 11-mercaptoundecanoic acid (MUA), thereby rendering the wire suitable for subsequent biofunctionalization. Figure 1B shows the practical single-wire sensor configuration, in which the γ-bent, solution-immersed segment defines the active sensing region, while the ends of the same continuous NiTi wire that remain outside the solution function as electrical contacts for EIS measurements.

As a proof of concept of this high-frequency dielectric sensing mechanism, γ-bent NiTi wire sensors achieved a detection limit of 64 CFU mL^−1^ for *E. coli* in sterile human urine, with a linear range of 10^2^–10^6^ CFU mL^−1^ within 45 min. To our knowledge, this is the first demonstration of superelastic NiTi microcracks mimicking self-healing behavior to function as metal-gap-metal transduction cavities for biosensing. These results define a new class of metallic flexible biosensors that combine mechanical robustness, simple fabrication, and clinically relevant analytical performance for point-of-care applications.

## 2. Materials and Methods

### 2.1. Materials

Superelastic NiTi wires (100 µm diameter) were purchased from Aksöz R&D Co. (Pamukkale, Türkiye) and cut into 4 cm segments. Hydrofluoric acid, nitric acid, and sodium hydroxide used for surface treatment were purchased from Apeks Group Co. (Bursa, Türkiye). Ethanol was purchased from Merck KGaA (Darmstadt, Germany). Phosphate-buffered saline (PBS, pH 7.4), 11-mercaptoundecanoic acid (MUA), N-(3-dimethylaminopropyl)-N′-ethylcarbodiimide (EDC), N-hydroxysulfosuccinimide (Sulfo-NHS), and bovine serum albumin (BSA) were obtained from Sigma-Aldrich (St. Louis, MO, USA). Escherichia coli ATCC 25922, Proteus mirabilis, and Klebsiella pneumoniae strains were supplied by the clinical microbiology laboratory. A rabbit polyclonal IgG antibody against *E.coli*, targeting surface antigens, was obtained from GeneTex, Inc. (Irvine, CA, USA; Cat: GTX13626). Sterile human urine was obtained as a commercially available research-grade product from Veritas Innovation, Inc. (Austin, TX, USA; Product Code: OH2050) and used as the negative control matrix for all electrochemical measurements. Data analysis and curve fitting were performed using OriginPro 2019b (OriginLab Corporation, Northampton, MA, USA). Further details on reagents and materials are provided in the Supplementary Material.

### 2.2. Preparation of NiTi Microcrack Transducers

Straight superelastic NiTi wires were cleaned in ethanol, etched with Kroll’s reagent for 2 min, rinsed with deionized water, and hydroxylated in 10 M NaOH at 60 °C for 30 min. After chemical treatment, each wire was bent into a γ-shaped configuration using a fixed holder (Figure 1B). This deformation generated stress-induced microcracks associated with martensitic transformation, whose internal gaps served as confined cavities that hosted the functional layers and the subsequently captured analytes. The γ-shaped constraint was maintained during all following functionalization and measurements to preserve the microcrack architecture.

### 2.3. Surface Modification

Wires were incubated overnight in ~3.2 mM ethanolic MUA solution to form thiolated self-assembled monolayers via Ni–S and Ti–O–S bonding. Carboxyl groups were activated in EDC/Sulfo-NHS solution for 60 min and briefly rinsed with PBS. *E. coli*-specific antibodies were incubated with the activated surface for 60 min, yielding covalently linked amide bonds. BSA blocking was applied solely for AFM studies to visualize protein retention and was not included in biosensing experiments.

### 2.4. Impedance Measurements

Impedance measurements were performed using a two-electrode configuration, where the γ-bent NiTi wire served as both working and counter electrodes. Measurements were conducted with a portable impedance analyzer (AIM-4300, Array Solutions, Texas, TX, USA) over the 0.01–10 MHz frequency range.

After antibody immobilization, the wire was placed in 150 µL sterile human urine to stabilize the baseline for 45 min. Bacterial suspensions (10^2^–10^7^ CFU mL^−1^) were then added without any washing step to avoid disturbing the stress-induced microcrack structure. Impedance spectra were collected at 15, 30, and 45 min.

ΔRct values were calculated from the real-axis intercepts of fitted Nyquist plots using a parabolic model in Origin. ΔRct was obtained from the change in the real-axis intercept between baseline and post-incubation spectra. The percentage change in charge-transfer resistance (ΔRct (%)) was calculated using:

(1) $$\Delta Rct\left(\%\right)=\frac{Rct,after-Rct,before}{Rct,before}\times 100$$

where *Rct,before* denotes the baseline resistance prior to bacterial exposure, and *Rct,after* corresponds to the value measured after incubation. All measurements were performed in triplicate.

### 2.5. Selectivity and Repeatability Tests

Selectivity was evaluated by comparing impedance responses from mixtures containing only non-target bacteria (*K. pneumoniae* + *P. mirabilis*, 10^5^ CFU mL^−1^ each) and mixtures additionally containing *E. coli* at the same concentration. Repeatability was assessed from triplicate measurements at each bacterial concentration (*n* = 3 per level), and the corresponding statistical metrics (mean, SD, SE, %RSD) are summarized in Table S1.

### 2.6. Characterization and Analysis

Surface chemistry and morphology were assessed using Fourier Transform Infrared (FTIR) spectroscopy (PerkinElmer, Waltham, MA, USA), Atomic Force Microscopy (AFM) (Veeco, Plainview, NY, USA), and Scanning Electron Microscopy (SEM) (Zeiss, Oberkochen, Germany). FTIR confirmed self-assembled monolayers (SAM) formation through characteristic COO^−^, CH_2_, and Ti–O–S bands (Figure S1). AFM quantified roughness changes across modification steps, revealing increased surface complexity consistent with successful functionalization (Figure S2). SEM further visualized the microcrack morphology and the resulting *E. coli* entrapment within stress-induced microcracks (Figure S3). Electrochemical validation of the surface modification and signal stability for the 250 µm wire (Figure S4) along with detailed instrumentation parameters, are provided in the Supplementary Material.

## 3. Results

The impedimetric behavior of the superelastic NiTi sensor stems from stress-induced martensitic transformation, which produces a heterogeneous surface morphology composed of non-cracked regions passivated by a native TiO_2_ layer and a network of reversible microcracks. This configuration enables the alloy to withstand substantial deformation without permanent damage and forms the basis for its sensing functionality [17,18].

**Figure 2:**
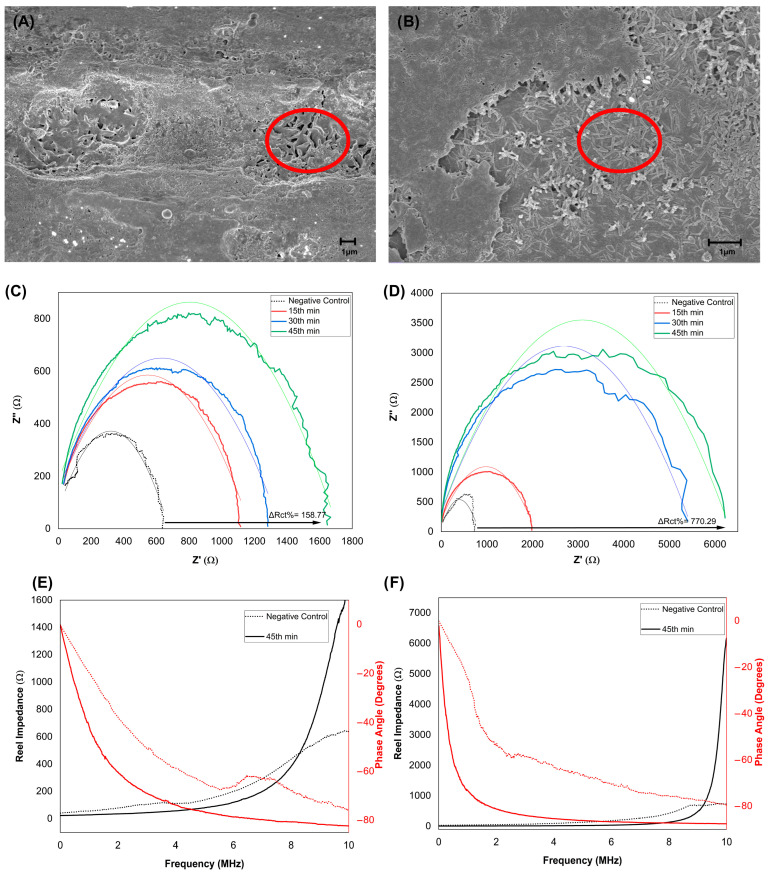
Morphological and electrochemical responses of the NiTi biosensor to *E. coli* in urine. (**A**,**B**) SEM images of sensor surfaces after exposure to 10^3^ CFU mL^−1^ (**A**) and 10^7^ CFU mL^−1^ (**B**), showing individual *E. coli* cells and cell clusters entrapped within martensitic microcracks (red circles). (**C**,**D**) Time-resolved Nyquist plots for 10^3^ CFU mL^−1^ (**C**) and 10^7^ CFU mL^−1^ (**D**), concentration-dependent increases in impedance. (**E**,**F**) Show the frequency-domain response at 10^3^ CFU mL^−1^ (**E**) and 10^7^ CFU mL^−1^ (**F**).

In the undeformed austenitic state, the NiTi surface exhibits homogeneous metallic character with negligible dielectric heterogeneity. Hence, contributions from microcrack-based capacitive elements are effectively absent under these conditions. Upon alkaline hydroxylation and mechanical deformation, however, the surface becomes chemically and structurally heterogeneous, with hydroxylated TiO_2_ regions and exposed Ni-rich domains within microcracks. These domains provide reactive sites for thiol-based surface functionalization. Self-assembled monolayers of MUA were chemisorbed onto the surface, forming Ti–O–S linkages at hydroxylated sites [19,20] and Ni–S bonds at metallic Ni sites (Figure 1A) [21,22]. The terminal carboxylic acid (–COOH) groups of MUA remained available for subsequent EDC/NHS crosslinking and antibody immobilization. Once embedded within the microcrack network, the MUA layer acts as a confined dielectric interface between conductive NiTi crack walls, effectively behaving as a thin parallel-plate capacitor. Exposure to analytes then modulated the local dielectric environment, producing measurable impedance changes driven by interfacial capacitance. The morphological entrapment of bacteria and the resulting electrochemical shifts of this mechanism are further detailed in Figure 2.

**Figure 3:**
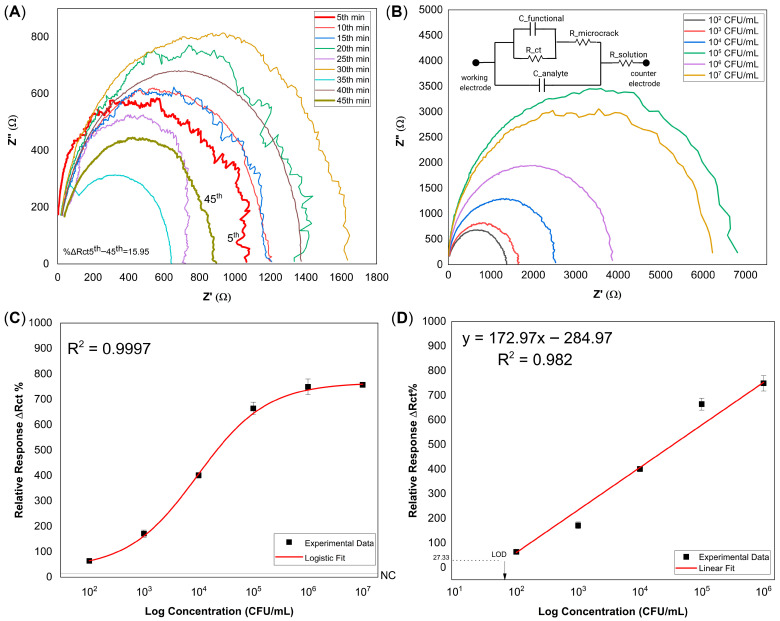
(**A**) Time-resolved Nyquist plots of a single NiTi microcrack sensor recorded in sterile human urine (negative control) at 5 min intervals over a 45 min period, showing signal stabilization and defining 45 min as the optimal incubation time. (**B**) Nyquist plots obtained after 45 min incubation with *E. coli* at concentrations from 10^2^ to 10^7^ CFU mL^−1^, each measured with a separate sensor, showing concentration-dependent impedance changes (ΔZ). The inset shows the equivalent circuit model. (**C**) Sigmoidal dose–response curve obtained by logistic fitting of the relative ΔRct response as a function of log[*E. coli*] concentration over 10^2^–10^7^ CFU mL^−1^ (*n* = 3). NC denotes the negative control. (**D**) Linear calibration curve constructed within the linear operating range of the sensor as a function of log[*E. coli*] concentration (10^2^–10^6^ CFU mL^−1^, *n* = 3). The LOD calculated from the linear fit was ~64 CFU mL^−1,^ corresponding to the intersection with the detection threshold (y = 27.33).

To aid interpretation of this behavior, a simplified equivalent circuit is included in the inset of Figure 3B solely as a conceptual illustration of the proposed impedance transduction mechanism and was not used for quantitative fitting or parameter extraction. This custom equivalent circuit topology departs from conventional Randles-type systems commonly applied to faradaic electrochemical interfaces. In classical Randles circuits, impedance is primarily governed by charge-transfer resistance and diffusion-related Warburg elements associated with mass-transport-limited electrochemical reactions. In contrast, the present NiTi electrode operates as a non-faradaic, capacitively dominated sensor, in which signal generation arises predominantly from dielectric changes within confined microcrack domains rather than from interfacial redox processes. While prior studies have investigated the electrochemical behavior of NiTi under mechanical loading and stress-induced martensitic reorientation [23], the present illustration additionally incorporates functional layers (C_functional_) and captured analytes (C_analyte_), introducing extra dielectric contributions. The capacitive elements associated with these confined regions (C_functional_ and C_analyte_) dominate the high-frequency response, consistent with dielectric modulation, whereas diffusion-related components such as Warburg impedance are absent.

Resistive pathways formed by stress-induced microcracks (R_microcrack_) describe heterogeneous conduction across transformed domains, while the solution resistance (R_solution_) accounts for the bulk electrolyte contribution. Together, these elements provide a qualitative framework for describing the transition from low-impedance metallic behavior in the austenitic state to a higher-impedance dielectric–metal interface following martensitic transformation and biofunctionalization.

### 3.1. Morphological and Electrochemical Characterization of the NiTi Biosensor

Scanning electron microscopy (SEM) was utilized to observe the interaction of *E. coli* with stress-induced microcracks on the NiTi sensor surface. Representative images at 10^3^ and 10^7^ CFU mL^−1^ (Figure 2A,B) show bacterial colonies entrapped within martensitic microcracks, highlighted in red circles. Increasing bacterial density was evident at higher concentrations, consistent with the concentration-dependent electrical response of the sensor.

SEM images show *E. coli* association with both the outer TiO_2_-covered surface and stress-induced microcracks, consistent with the dual-surface functionalization strategy illustrated in Figure 1. High-magnification SEM images at 10^2^, 10^3^, and 10^7^ CFU mL^−1^ are provided in Figure S3; the 10^2^ CFU mL^−1^ images represent the lowest experimentally evaluated concentration and show early-stage bacterial localization within microcrack features. Colonies were predominantly localized within microcrack regions.

Electrochemical impedance spectroscopy (EIS) was then used to monitor the dynamic binding behavior of the biosensor across a wide frequency range (0.01–10 MHz). The impedimetric response was evaluated through Nyquist plots, where the real part of impedance (Z′) is plotted on the x-axis and the imaginary part (Z″) on the y-axis [24]. Time-resolved Nyquist plots (Figure 2C,D) showed gradual increases in impedance at 10^3^ CFU mL^−1^ over 15–45 min, indicative of progressive antibody–antigen binding. In contrast, exposure to 10^7^ CFU mL^−1^ produced a rapid rise within the first 15 min, followed by signal saturation. Frequency-domain spectra (Figure 2E,F) exhibited pseudocapacitive behavior, with phase angles approaching −80° at high frequencies [23].

### 3.2. Analytical Performance of the NiTi Biosensor

EIS was employed to monitor each step of the surface modification process (Figure S3A). Distinct impedance changes were observed following NaOH activation, MUA functionalization, EDC/NHS crosslinking, and antibody immobilization, confirming successful functionalization. The thinner 100 μm sensor reached a stable baseline, whereas the 250 μm wire showed more variable profiles, highlighting the effect of wire thickness on stability (Figure S4B). Surface roughness changes accompanying these modification steps were further verified by AFM (Figure S2).

Following final surface modification, all NiTi microcrack sensors were first monitored in sterile human urine for 45 min, during which impedance spectra were recorded at 5 min intervals to assess baseline stability under negative-control conditions. Figure 3A shows a representative time-resolved Nyquist response obtained from a single sensor. This pre-stabilization step confirmed equilibration of the microcrack-associated impedance response and defined 45 min as the optimal incubation time prior to bacterial exposure. After stabilization, independent NiTi sensors were incubated with *E. coli* suspensions at concentrations ranging from 10^2^ to 10^7^ CFU mL^−1^ for 45 min. Due to intrinsic wire-to-wire variations in microcrack morphology, each sensor exhibited a distinct baseline impedance response in urine. To account for this variability, the impedance spectrum measured after *E. coli* incubation was baseline-corrected by subtracting the corresponding negative-control response obtained from the same sensor, yielding ΔZ = Z_FINAL_ − Z_NC_. Figure 3B shows representative baseline-corrected Nyquist plots selected from triplicate measurements for each concentration, illustrating how microcrack morphology influences the concentration-dependent impedance response. Although the incremental change in charge-transfer resistance (ΔRct) increased with *E. coli* concentration, the absolute semicircle diameters were not strictly monotonic due to inherent microcrack heterogeneity among individual sensors. The equivalent circuit depicted in Figure 3B is provided as a simplified conceptual representation of the proposed impedance modulation mechanism and was not used for quantitative fitting or for deriving ΔRct values. The corresponding dose–response behavior, obtained by averaging ΔRct values from three independent sensors per concentration (*n* = 3), exhibited an overall sigmoidal trend across the full concentration range of 10^2^–10^7^ CFU mL^−1^ (Figure 3C), consistent with the characteristic saturation behavior of antibody–antigen interactions in immunoassay systems, and was well described by a logistic fit (R^2^ = 0.9997). At high analyte levels, the response approached saturation, resulting in deviation from linearity at 10^7^ CFU mL^−1^. Therefore, quantitative analysis was restricted to the linear operating range of the sensor (10^2^–10^6^ CFU mL^−1^), in accordance with established analytical practices for immunoassay-based sensing systems. Within this linear operating range, the data were subjected to linear fitting to construct the calibration curve and enable quantitative analysis and limit of detection (LOD) estimation (Figure 3D). The LOD was determined using an IUPAC-compliant threshold intersection approach, in which the threshold signal was defined as the mean negative-control response plus three times its standard deviation [25]: $y_{\left\{LOD\right\}}=\bar{{\left\{x\right\}}_{\left\{NC\right\}}}+3\sigma_{\left\{NC\right\}}$. This analysis yielded a calibration curve with good linearity (R^2^ = 0.982) and an estimated LOD of approximately 64 CFU mL^−1^.

Selectivity was evaluated by exposing the NiTi biosensor to a mixture of non-target bacteria, specifically *Klebsiella pneumoniae* and *Proteus mirabilis* at a concentration of 10^5^ CFU mL^−1^. This interference mixture yielded a negligible mean response (ΔRct = 8.75 ± 1.77 %, *n* = 3). In contrast, the subsequent inclusion of *E. coli* (10^5^ CFU mL^−1^) into the mixture resulted in a pronounced increase (ΔRct = 434.72 ± 44.69%, *n* = 3). Statistical analysis confirmed that the difference between groups was significant (*p* < 0.001, one-way ANOVA).

## 4. Discussion

This study introduces a fundamentally new sensing architecture by transforming superelastic NiTi wires into label-free impedimetric biosensors. By exploiting stress-induced martensitic microcracks as confined cavities that host the functional interfacial layers, the system achieves molecular recognition purely through impedance changes, without faradaic reactions or diffusion-limited processes. Notably, no prior biosensor has harnessed the intrinsic microstructural changes in NiTi in this way; for the first time, its stress-induced microcracks are repurposed as confined transduction cavities whose dielectric interiors form the active high-frequency sensing domain, eliminating the need for engineered nanostructures or external capacitive layers.

The sensing principle relies on microcracks mimicking self-healing generated during stress-induced martensitic transformation [10,26,27,28]. These cracks open under stress and close upon unloading, forming reversible confined cavities that host functional layers and captured analytes [29]. While microcracks provide the physical transduction framework, selective molecular recognition is achieved through stepwise surface functionalization with thiolate SAMs, EDC/NHS chemistry, and antibody immobilization [30,31]. FTIR spectra (Figure S1) further confirmed thiol anchoring by the disappearance of the –SH stretching band [32].

AFM measurements confirmed a progressive increase in surface roughness across these steps (Figure S2), supporting the formation of a heterogeneous, biofunctional interface. Notably, regarding the spatial distribution of bacterial capture, this functionalization is not strictly limited to the microcrack interiors. As illustrated in the workflow (Figure 1), superelastic deformation generates a chemically heterogeneous surface where exposed nickel-rich sites facilitate direct Ni–S bonding [21,28,32,33], while hydroxylated TiO_2_ regions support Ti–O–S linkages [19,20]. SEM imaging (Figure S3) confirms this dual mechanism: while *E. coli* cells adhere to the outer oxide layer at lower concentrations (10^2^ CFU mL^−1^), they preferentially localize within the confined microcrack domains at higher loads. Although binding occurs in both regions, the impedimetric signal is dominated by the microcracks. This is because the confined geometry of the cracks creates a “parallel-plate” capacitor effect with a very narrow dielectric spacing (d), which, according to C = εA/d, results in a significantly higher capacitance change compared to the intact surface. Thus, while the outer surface contributes to capture efficiency, the functionalized microcracks act as the primary contributors of high-frequency dielectric signal modulation.

It is crucial to distinguish this sensing mechanism from the thermal shape memory effect often associated with NiTi alloys. While shape memory applications rely on temperature variations to drive phase transformation, our sensor operates in the superelastic regime, where martensitic transformation is induced purely by mechanical stress at a constant temperature [14]. Because the utilized NiTi wires possess an austenite finish temperature (A_f_) of approximately 15 °C, the material remains in the austenitic phase at room temperature. This ensures that the observed microcracks are generated exclusively by mechanical loading without requiring external heating, thereby preserving the structural integrity of temperature-sensitive biological analytes. Notably, superelasticity is an intrinsic property bounded by the transformation temperatures. Since the operational window requires T > A_f_ [10,14], the current sensor design is optimized for environments above 15 °C, encompassing typical laboratory and clinical settings. For potential applications in colder environments, the alloy composition would need to be tuned to lower the A_f_.

Unlike conventional impedimetric electrodes, this system exhibits a strong pseudocapacitive component at higher frequencies (0.01–10 MHz). Notably, phase angles reach approximately −80° even at low concentrations of 10^2^ CFU mL^−1^ and shift toward more negative values as the bacterial load increases, indicating a transition toward ideal capacitive behavior (Figure 2E,F) [23]. Previous NiTi electrochemical models primarily described passive film resistance and charge transfer kinetics [23,27,28,34,35]. In contrast, while bulk NiTi behaves as a standard conductor at low frequencies, our results confirm that the functionalized microcracks act as dominant capacitive elements specifically under high-frequency interrogation. This frequency-dependent transition proves that the observed signal is not merely a change in surface resistance but a distinct dielectric modulation arising from the confined biomolecular interfaces within the cracks. This behavior links the microcrack architecture directly to dielectric modulation, providing a previously unreported mechanism for signal generation in NiTi-based systems.

Stepwise modification of the NiTi surface produced predictable shifts in charge transfer resistance, consistent with changes in surface chemistry (Figure S4A). The initial increase in Rct after NaOH treatment reflected the formation of a resistive hydroxide layer [36,37]. Subsequent MUA functionalization, EDC/NHS crosslinking, and antibody immobilization progressively decreased Rct as molecular order improved, establishing a stable bio-recognition interface. Although the accumulation of insulating organic layers typically increases impedance, the observed progressive decrease across these steps is primarily attributed to the mechanical equilibration of the stress-induced microcracks. In the γ-bent configuration, the sensing region remains under constant elastic strain. Upon liquid exposure and functionalization, this microcrack network undergoes a dynamic stabilization phase, where subtle geometric reconfigurations allow the interfacial charge distribution to reach a steady state. As evidenced by the time-dependent kinetics (Figure 3A, separate sensor), this impedance drop represents a transient mechanical relaxation toward a stable baseline, ensuring that the final sensing signals reflect analyte binding rather than structural drift.

Sensor stability depended significantly on wire diameter and mechanical design. Experimental results demonstrated that 100 μm NiTi sensors produced consistent Nyquist profiles that stabilized within minutes (Figure 3A), in contrast to 250 μm wires displayed inconsistent signals without a stable baseline (Figure S4B). A similar diameter dependence has been reported in independent studies on NiTi strain sensors [38], suggesting that thinner wires inherently form narrower, more homogeneous microcrack networks, while thicker wires develop more dispersed domains [39,40]. Since NiTi maintains superelasticity down to ~100 nm [41,42,43], further miniaturization is expected to enhance sensitivity and integration into portable devices. To ensure the reliability of the presented data, a standardized urine matrix was strategically utilized in this study. This approach eliminated confounding variables from patient-specific ionic fluctuations, allowing us to characterize more clearly the intrinsic impedance response driven by bacterial binding. Regarding the sensor fabrication, commercially available 100 µm diameter NiTi wires were selected to demonstrate the broad accessibility and practical applicability of the proposed architecture. While this configuration provided reliable analytical performance suitable for proof-of-concept, future clinical translation efforts will focus on further miniaturization (<100 µm). Scaling down to finer diameters is expected to enhance batch-to-batch uniformity and reproducibility, facilitating mass production for point-of-care applications.

The sensor exhibited a characteristic dose–response profile governed by the saturation kinetics of antibody–antigen interactions. The curve followed a sigmoidal shape (R^2^ = 0.9997) across the full tested range (10^2^–10^7^ CFU mL^−1^), matching the behavior of high-affinity immunosensors (Figure 3C). Within the linear dynamic range (10^2^–10^6^ CFU mL^−1^), the sensor showed strong linearity (R^2^ = 0.982), yielding LOD of 64 CFU mL^−1^ (Figure 3D). Repeatability analysis (Table S1) further confirmed consistent responses across independent measurements. Notably, the calculated LOD lies below the lowest experimentally tested concentration (10^2^ CFU mL^−1^), underscoring the high sensitivity of the microcrack-enhanced dielectric transduction. Crucially, no Warburg-type diffusion elements were observed even at high concentrations, indicating that signal modulation is governed by interfacial dielectric changes rather than mass transport [44,45]. Finally, selectivity tests confirmed that non-target bacteria (*K. pneumoniae* and *P. mirabilis*) induced negligible impedance changes compared to *E. coli*. Although this work focused on *E. coli*, the modular surface chemistry can be readily adapted to other biorecognition elements, extending the sensing architecture to proteins, toxins, or viral targets.

To contextualize these findings, Table 1 compares the present NiTi platform with representative flexible biosensors reported in the recent literature. The comparison highlights that polymer-based systems often depend on engineered nanostructures or composite layers to achieve flexibility, whereas the current work uniquely exploits the intrinsic solid-state transformation of a metallic alloy. The NiTi architecture therefore bridges the gap between polymeric flexibility and metallic robustness, introducing a recyclable, corrosion-resistant, and mechanically adaptive alternative for flexible biosensing.

While lacking the geometric regularity of lithography, stress-induced microcracks offer a unique dynamic advantage that static etching cannot replicate: the ability to mimic self-healing behavior via superelastic closure. By utilizing these defects, which are conventionally dismissed as structural failures, we leverage their exposed nickel-rich interiors to create confined cavities that uniquely enable high-frequency dielectric transduction. This paradigm shift converts intrinsic disorder into a non-faradaic functional utility, transforming the passive alloy into a chemomechanically responsive transducer and establishing a scalable blueprint for flexible biosensing.

## 5. Conclusions

This work demonstrates that superelastic NiTi can serve as a mechanically flexible, low-cost and label-free impedimetric transducer. By exploiting stress-induced martensitic microcracks as confined transduction cavities, we established that their functionalized interiors form an active high-frequency sensing domain, generating measurable impedance changes driven by dielectric modulation rather than faradaic reactions. The developed sensor enabled rapid and specific detection of *E. coli* in urine with a LOD of 64 CFU mL^−1^, utilizing simple bending mechanics and standard wet-chemistry processing. These findings highlight the potential of NiTi microcrack architectures for developing simple, robust, affordable and portable point-of-care biosensors.

## 6. Patents

An international patent application (PCT/TR2024/050849) related to the findings of this study was filed on 18 July 2024.

## Figures and Tables

**Table 1:** Comparison of representative flexible biosensors categorized by substrate type (polymeric, metallic, or hybrid) and mechanical flexibility origin.

Sensor Material	Flexibility Type	LOD	Linear Range	Detection Time (min)	Matrix	POCT Suitability	Ref
Ferrocene-based PVA/SA/PEI-Fc hydrogel	Polymeric (Hydrogel)	Not reported	Real time	Real time	PBS; cell culture media (L929, RAW 264.7)	Yes (in vitro validated)	[46]
PDMS/PET–Au/MoS_2_–GOx nanofilm	Polymeric (PET)	0.10 nM	10–500 nM	30 min	Glucose in PBS	Yes (in vitro validated)	[47]
AuNPs-LIG interdigitated electrode (flexible)	Polyimide substrate	10^2^ CFU mL^−1^	10^2^–10^8^ CFU mL^−1^	30 min	*E. coli* in PBS	Not target	[48]
Silk fibroin flexible substrate + PEDOT:PSS + anti-VEGF	Polymeric (organic)	22–25 pg mL^−1^	1 pg mL^−1^–10 mL^−1^	15 min	PBS, serum, artificial urine	Yes (in vitro validated)	[49]
AlN thin film on PEN substrate (SAW)	Polymeric (PEN)	6.5 × 10^5^ CFU mL^−1^	10^6^–10^8^ CFU mL^−1^	90 min	*E. coli* in water	Moderate (High LOD)	[50]
AuNP-SiO_2_-QDs nanocomposite on LFIA strip	Polymeric LFIA substrate	IL-6: 4.7 pg mL^−1^; PCT: 29.5 pg mL^−1^	0.1–100 ng mL^−1^	15 min	Human plasma (clinical)	Yes (clinically validated)	[51]
Ag-decorated magnetic nanobeads on paper-based aptasensor	Paper substrate	DPV: 90; EIS: 8.09 CFU mL^−1^	DPV: 10^2^–10^8^; EIS: 10^1^–10^9^ CFU mL^−1^	40 min	Food	Yes (in vitro validated)	[52]
Stretchable gold fiber textile	Elastomeric textile	0.137 mM	0–30 mM	Real time	Lactate in artificial sweat	Yes (in vitro validated)	[53]
CNT/PDDA + AuNP multilayer on Ga-based liquid metal	Intrinsically soft liquid metal core (ultrasoft)	23 nM	25 nM–1 µM	Real time	PBS/ACSF (in vitro neurochemical buffer)	Not target	[54]
Ti-doped MoTe_x_ film EGFET	Thin-film hybrid	5 × 10^−3^ pg mL^−1^	10^−2^–10^5^ pg mL^−1^	Real time	BNP in serum	Yes (clinically validated)	[55]
NiS/Ni-foam	Metallic foam	159 μM (A), 147.6 μM (G), 16.8 μM (T), 45.9 μM (C)	200–1000 μM (A,G); 50–500 μM (T,C)	Real time	Calf thymus DNA	Yes (in vitro validated)	[56]
Superelastic NiTi alloy	Intrinsic solid-state microcracks	64 CFU mL^−1^	10^2^–10^6^ CFU mL^−1^	45 min	*E. coli* in sterile human urine	Yes (proof of concept)	This work

## Data Availability

The data presented in this study are available from the corresponding author upon request.

## Supplementary Materials

The following supporting information can be downloaded at https://www.mdpi.com/article/10.3390/bios16010054/s1, File S1: Extended Materials and Methods, Figures S1–S4, and Table S1. Figure S1: FTIR spectra of bare, NaOH-treated, and MUA-functionalized NiTi surfaces; Figure S2: AFM images showing surface morphology changes during sequential modification steps; Figure S3: SEM micrographs revealing the microcrack morphology and surface-bound bacteria at concentrations of 10^2^, 10^3^, and 10^7^ CFU mL^−1^; Figure S4: Nyquist plots demonstrating the stepwise surface modification process (A) and the stability profile of 250 µm NiTi electrodes in sterile human urine (B). Table S1: Calibration repeatability results including mean, SD, SE, and %RSD values. The Supplementary Materials also cite Refs. [20,21,22,33,36,37,57,58,59,60,61,62,63,64] for methodological background and supporting literature.
