# Supplementary material for: Metallic Flexible NiTi Wire Microcrack Transducer for Label-Free Impedimetric Sensing of Escherichia coli
Source: Biosensors (Basel). 2026 Jan 10;16(1):54. doi: 10.3390/bios16010054 (PMC12839385; doi:10.3390/bios16010054)
Supplement: Supplementary file 1 [file biosensors-16-00054-s001.zip › FILE S1/SUPPLEMENTARY MATERIAL.pdf]

SUPPORTING INFORMATION

Metallic Flexible NiTi Wire Microcrack Transducer for Label-Free Impedimetric Sensing of E. coli

Gizem ÖZLÜ TÜRK [1,2,3], Mehmet Çağrı SOYLU [1,2]*

[1] Biological and Medical Diagnostic Sensors Laboratory (BioMeD Sensors Lab), Department of Biomedical Engineering, Faculty of Engineering, Erciyes University, Kayseri 38030, Türkiye

[2] Biomedical Engineering Program, Graduate School of Natural and Applied Sciences, Erciyes University, Kayseri 38280, Türkiye

[3] Biomedical Device Technologies Program, Department of Electronics and Automation, Ercis Vocational School, Van Yuzuncu Yil University, Van 65400, Türkiye

*Corresponding Author's Email: mcsoylu@erciyes.edu.tr

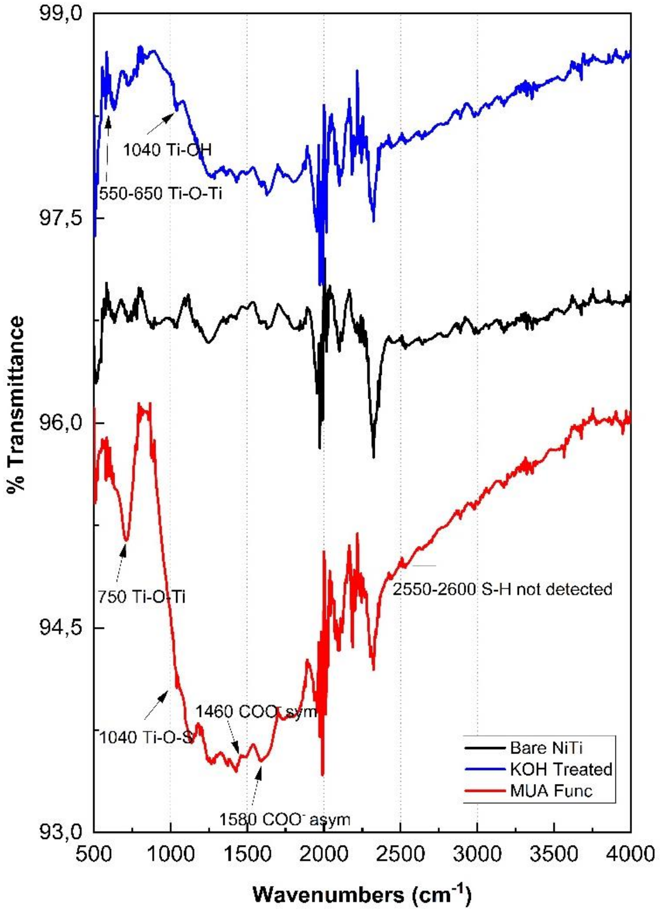

Figure S1. FTIR spectra of bare, NaOH-treated, and MUA-functionalized NiTi surfaces. Characteristic peaks confirm sequential modification: COO^- stretches (~1583, 1459 cm^{-1}), CH_2 vibrations (~2921, 2851 cm^{-1}), and Ti–O–S bonding (~1040 cm^{-1}). The disappearance of the S–H band (2550–2600 cm^{-1}) indicates successful chemisorption of thiols via Ni–S interaction.

As shown in Figure S1, the FTIR spectrum of the MUA-functionalized NiTi surface exhibits several distinct absorption bands that confirm the successful formation of a thiol-based self-assembled monolayer (SAM). Both bare and NaOH-treated NiTi surfaces exhibit a broad band between 450 and 750 cm^{-1}, corresponding to Ti–O and Ti–OH stretching vibrations, indicative of hydroxylated TiO_2 and effective surface activation prior to SAM formation[1] . Following MUA-functionalization, characteristic peaks were observed at ~1040 cm^{-1} (Ti–O–S)[2], 1459–1468 and 1583–1593 cm^{-1} (symmetric/asymmetric COO^- stretching), and 2850–2920 cm^{-1} (–CH_2– vibrations)[1, 3]. The disappearance of the S–H stretching band at 2550–2600 cm^{-1} confirmed thiol chemisorption via Ni–S bonding. The observed Δv values were consistent with bidentate coordination between carboxylate groups and the metal oxide surface [3].

Atomic force microscopy (AFM) imaging was performed at four key stages (Figure S2): (i) the unmodified martensitic-phase NiTi surface after Kroll's reagent, and deionized water and ethanol

treatment; (ii) following surface hydroxylation; (iii) after self-assembled monolayer (SAM) formation using MUA and EDC/Sulfo-NHS chemistry; and (iv) after BSA immobilization.

The initial AFM image (Figure. S2A) was acquired from a localized martensitic region exhibiting surface-level features associated with early-stage stress-induced transformation. The relatively low roughness values (Ra = 6.27 nm, Rmax = 78.09 nm) reflect a partially activated morphology, without prominent trench-like fissures. Despite this modest topography, the disruption of the native TiO_2 layer enables exposure of chemically active Ni-enriched sites that facilitate thiol chemisorption. This allows robust SAM formation through Ni–S bonding, even in the absence of deep crack penetration [3, 4].

Quantitative AFM analysis revealed a stepwise increase in surface roughness following chemical modification (Figure S2). The unmodified NiTi surface exhibited a baseline Ra of 6.27 nm and Rmax of 78.09 nm, reflecting its native martensitic microcrack morphology, as further corroborated by SEM imaging. Alkaline hydroxylation using Kroll's reagent elevated the roughness to Ra = 19.13 nm, Rmax = 217.98 nm by partially disrupting the TiO_2 layer and exposing nickel-rich domains. These sites enabled efficient thiol chemisorption of carboxyl-terminated MUA molecules, which further increased the surface roughness to Ra = 53.29 nm, Rmax = 499.09 nm. Unlike atomically flat substrates such as gold, the inherent topographical complexity of NiTi leads to a less ordered SAM structure and elevated nanoscale roughness [5]. Covalent immobilization of BSA via EDC/Sulfo-NHS crosslinking led to final roughness of Ra = 77.52 nm, Rmax = 820.80 nm, consistent with the formation of a protein layer through amide bond formation between $–NH_2$ groups on BSA and activated –COOH groups of MUA[6].

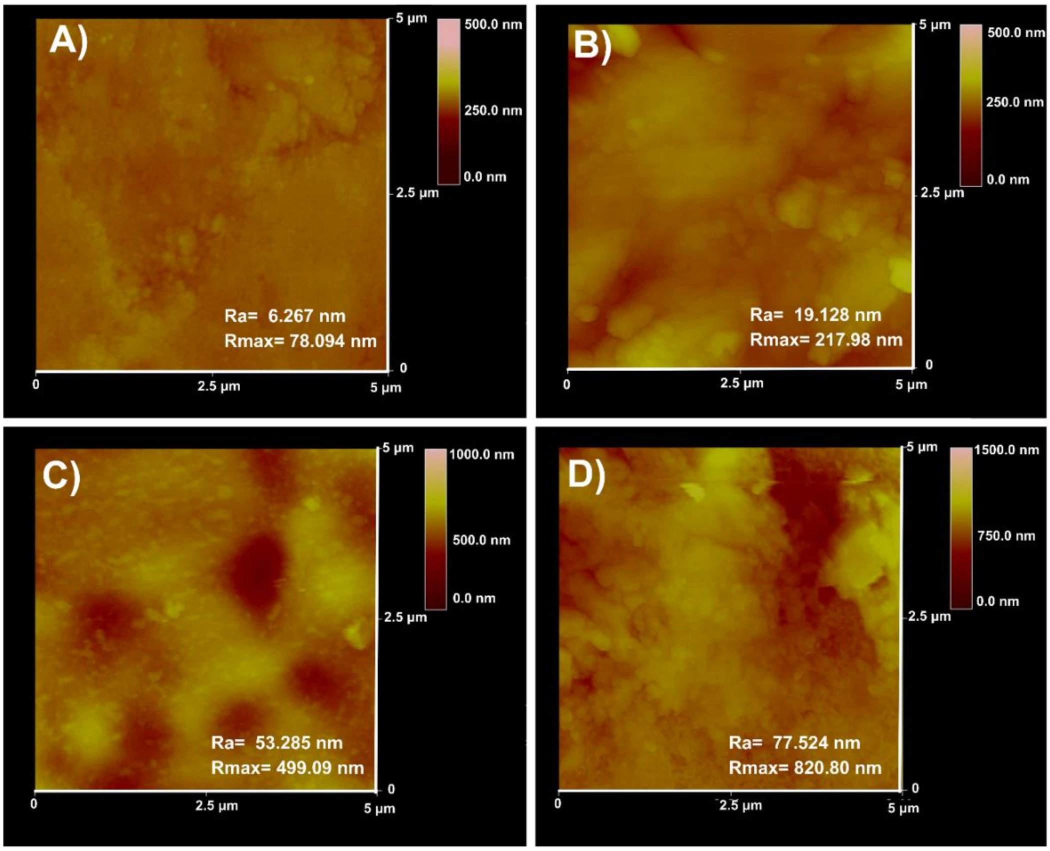

Figure S2. AFM images (5 µm × 5 µm) showing the surface morphology of NiTi at key stages of modification: (a) unmodified surface; (b) after hydroxylation; (c) after MUA+EDC/Sulfo-NHS functionalization; and (d) after BSA blocking

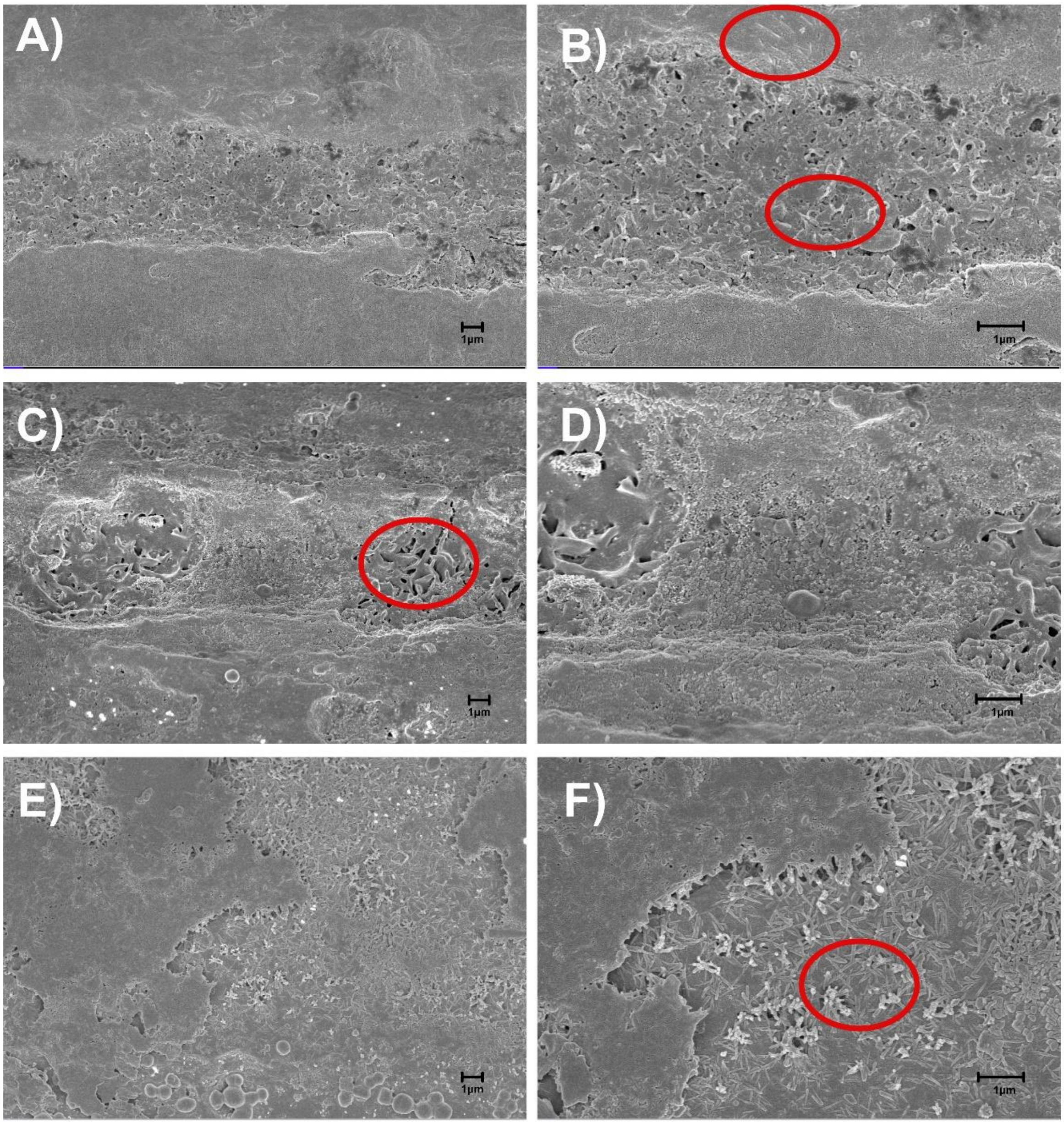

Figure S3. SEM images of *E. coli* attachment on the NiTi sensor surface at different bacterial concentrations. Panels A–B show representative images at 10^2 CFU/mL, where bacterial cells are observed both within stress-induced microcracks and on the outer TiO_2-covered surface. Panels C–D correspond to 10^3 CFU/mL, showing increased bacterial localization within microcrack regions. Panels E–F show images at 10^7 CFU/mL, where dense bacterial coverage is observed, with colonies predominantly occupying microcrack domains. Red circles highlight representative bacterial clusters.

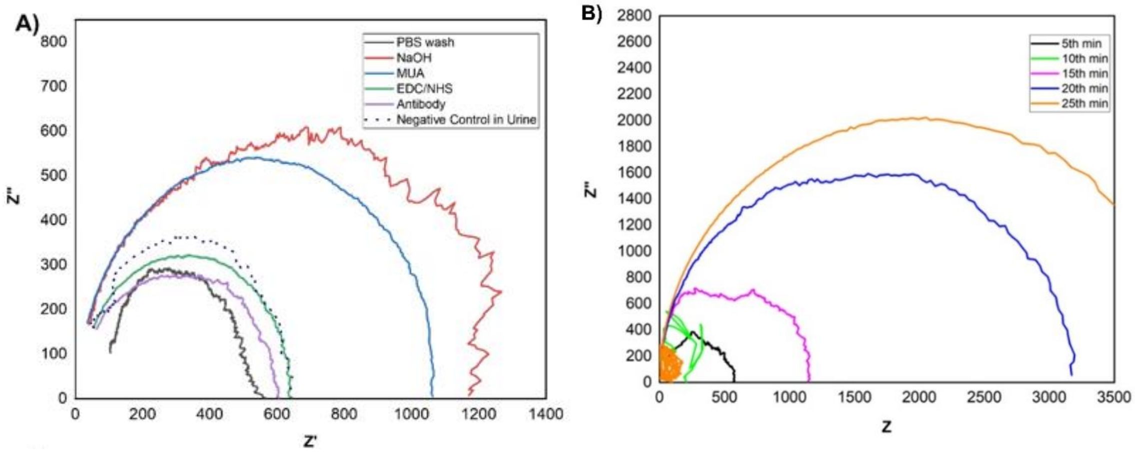

Figure S4. a) Nyquist plots of a single NiTi wire recorded after sequential surface modification: NaOH activation, MUA functionalization, EDC/NHS crosslinking, antibody immobilization, PBS wash, and negative control in urine. **b)** Nyquist plots of a 0.25 mm NiTi electrode in sterile urine (negative control), recorded every 5 minutes up to 25 minutes. Both samples underwent the same surface modification steps and were measured under identical conditions, differing only in wire thickness and total measurement duration.

1.1. Materials and Methods

Superelastic NiTi wire (2 m length, 100 μm diameter, composed of 55.79 wt% Ni, 44.1 wt% Ti, and 0.11 wt% other elements) was manufactured in a straight configuration by Aksöz R&D Co. (Pamukkale, Türkiye). Hydrofluoric acid (HF, 70% w/w; CAS: 7664-39-3), nitric acid (HNO_3, 55% w/w; CAS No: 7697-37-2), and sodium hydroxide (NaOH, pellets; CAS: 1310-73-2) were obtained from Apeks Group Co. (Bursa, Türkiye). As required by the Kroll etching protocol, the concentrated HF and HNO_3 solutions were diluted to 40% (w/w) with deionized water prior to application on the NiTi surface. Deionized water (DIW) was supplied by the Environmental Engineering Laboratory of Erciyes University (Kayseri, Türkiye). Ethanol (≥99.5%) was obtained from Merck KGaA (Darmstadt, Germany; Cat: 1.00983.2500). 11-Mercaptoundecanoic acid (MUA; Cat: 450561), N-(3-Dimethylaminopropyl)-N′-ethylcarbodiimide hydrochloride (EDC; Cat: E7750), N-hydroxysulfosuccinimide sodium salt (Sulfo-NHS; Cat: 56485), phosphate-buffered saline (PBS, pH 7.4; Cat: P3813) and bovine serum albumin (BSA; Cat: A9647) were purchased from Sigma-Aldrich (St. Louis, MO, USA).

The *Escherichia coli* ATCC 25922 strain (standardized to 0.5 McFarland), *Proteus mirabilis*, and *Klebsiella pneumoniae* were obtained from the Central Laboratory of Erciyes University Hospitals (Kayseri, Türkiye). A rabbit polyclonal IgG antibody against *E.coli*, targeting surface antigens, was obtained from

GeneTex, Inc. (Irvine, CA, USA; Cat: GTX13626). Human urine samples, confirmed to be negative for toxicological substances, were obtained from Veritas Innovation, Inc. (TX, USA; Product Code: OH2050).

The AIM-4300 Antenna Analyzer (Array Solutions, Sunnyvale, TX, USA; frequency range: 5 kHz to 300 MHz), used as a two-electrode electrochemical impedance spectroscopy (EIS) measurement device, was employed to record impedance spectra of the sensors.

1.2. Sensor Fabrication

Straight NiTi wires (100 μm in diameter) were cut into 4 cm segments. The wire samples were first cleaned by immersing them in ethanol for 2 minutes. This was followed by surface etching using Kroll's reagent (2 mL hydrofluoric acid [40%] + 1 mL nitric acid [40%] + 497 mL deionized water) for 2 minutes, and then rinsed in deionized water for an additional 2 minutes. Hydroxylation was subsequently applied to the wires.

Following this chemical treatment, each sample was shaped into a γ-bent configuration using a simple custom setup. The γ-shape induced localized martensitic transformation due to mechanical deformation, which in turn generated surface microcracks. These microcracks served two critical roles: they acted as confined dielectric regions essential for impedimetric sensing and exposed nickel-rich zones beneath the native oxide layer, enabling selective chemisorption of thiol-terminated molecules [4, 7, 8].

All NiTi wires were bent manually but consistently into a γ-configuration using the same mold geometry to ensure uniform curvature and deformation site. No annealing was performed, as self-healing martensitic microcracks form spontaneously under superelastic bending regardless of the applied bending magnitude.
To prepare the setup, the end of a plastic micropipette tip was trimmed, and two small holes were made near the narrow end using a fine needle. The NiTi wire was passed through both holes, creating a stable bent shape between the entry and exit points. This configuration held the wire firmly in place while keeping both ends exposed for electrical connection in a two-electrode EIS setup. The micropipette was then placed into a vertically cut Eppendorf tube, with the cap positioned at the bottom, serving as a non-contact container during all surface treatments and measurements. Liquid exchanges were performed by pipetting from the edge of the tube without disturbing the wire, ensuring the integrity of the microcrack network.

1.3. Sensor Functionalization

Hydroxylation: The samples were incubated at 60°C for 30 minutes in a 10 M NaOH solution prepared with DIW. After incubation, they were rinsed in DIW for 2 minutes [9, 10]. All treatments up to this

point were carried out with the wire in its linear, undeformed state. All subsequent surface modifications, including functionalization and biosensing steps, were performed in the gamma (ɣ) configuration to maintain mechanical constraint and preserve the microcrack architecture.

Self-Assembled Monolayers Functionalization: An ethanol solution containing 7 mg of MUA per 10 mL was prepared and evenly distributed to individual samples. The containers were then placed inside a sealed large chamber. Within the martensitic-phase microcracks, the exposed Ni-rich surfaces enabled the formation of stable Ni–S bonds through direct chemisorption of the thiol groups present in MUA, thereby establishing a robust self-assembled monolayer foundation for subsequent biofunctionalization (Figure S1). The samples were incubated overnight (≥14 hours) and subsequently rinsed in ethanol for 2 minutes to remove unbound residues [11].

EDC/Sulfo-NHS Crosslinking: A solution of 5 mg EDC, 5 mg Sulfo-NHS, and 1 mL sodium phosphate buffer (PBS, pH 7.4) was vortexed. The sensors were immersed for 60 minutes at room temperature. Excess reagents were removed by a brief 2-minute rinse in PBS. In this reaction, EDC activates surface carboxyl groups into an O-acylisourea intermediate, which is stabilized by Sulfo-NHS through formation of an NHS ester. This ester undergoes nucleophilic substitution by primary amines on the antibody, yielding a covalent amide bond and enabling robust antibody immobilization within the microcrack domains.

Antibody Modification: For each sensor, 180 µL of PBS was mixed with 20 µL of amine-active *E. coli* antibody solution (10^{15} particles/mL), and the sensors were incubated in the resulting mixture for 60 minutes. The amine-active antibodies were covalently linked via stable amide bonds. At the end of the procedure, the sensors were washed three times with PBS to remove unbound antibodies.

BSA Blocking: Following antibody immobilization, the sensors were incubated with 1% bovine serum albumin (BSA) in PBS for 45 minutes at room temperature to minimize non-specific adsorption and assess surface functionality. AFM analysis confirmed the retention of BSA on the modified surface. To avoid potential steric hindrance of the target bacteria, BSA was used only for AFM characterization and not included in any biosensing protocol [12].

1.4. Characterization of Functionalization

Fourier Transform Infrared (FTIR) Spectra were acquired on a 400 FT-IR/FT-FIR Spectrometer with Spotlight 400 Imaging System (PerkinElmer, Waltham, MA, USA) using diamond ATR mode. Measurements were performed in the 4,000–600 cm^{-1} range at a resolution of 4 cm^{-1}, with 32 co-added scans collected at room temperature.

Surface morphology and topographical changes were evaluated using a Multimode 8 AFM system (Veeco, Plainview, NY, USA) operated in tapping mode. Scans were performed over a 5 μm × 5 μm area at room temperature. Images were acquired for three representative conditions: bare NiTi, MUA + EDC/Sulfo-NHS modified surfaces, and BSA-coated layers. Roughness parameters including Ra (average roughness) and Rmax (maximum peak-to-valley height) were extracted for each condition.

E. coli entrapment within stress-induced martensitic microcracks on the NiTi sensor surface was visualized using a Gemini 500 SEM system (Zeiss, Oberkochen, Germany). Images were acquired at multiple magnifications for representative samples exposed to varying bacterial concentrations. Mechanically shaped γ-bent geometries were retained throughout the sample preparation and SEM imaging procedures to preserve the stress distribution relevant for microcrack formation.

1.5. Detection Set-up

Two-electrode configuration: Electrochemical impedance spectroscopy (EIS) measurements were performed using a two-electrode configuration with the AIM-4300 Antenna Analyzer. The γ-bent NiTi wire served as both the working and counter electrodes. The central segment of the wire, which had been chemically modified with SAMs and antibodies, functioned as the capacitive sensing interface. In contrast, the unmodified terminal regions retained their native conductivity and provided direct electrical contact with the measurement probes.

Sensor Mounting and electrical contact: Each functionalized NiTi wire was positioned inside a custom-designed container that preserved the γ-bent deformation throughout the experimental procedure. This container was specifically designed to apply an appropriate mechanical constraint to the wire, enabling the generation of martensitic microcracks under stress and facilitating the emergence of a stable capacitive response. The wire ends were connected to the EIS device probes before antibody immobilization and remained connected throughout all functionalization and detection steps. This configuration ensured mechanical stability and minimized any physical disturbance that might otherwise alter the stress-induced microcrack structure.

Liquid exchange method: To prevent disruption of the microcrack geometry or dislocation of the functional layers, all washing and liquid addition steps were carefully conducted using a micropipette directed along the inner wall of the container. At no point was the sensor surface directly contacted by

instruments or disturbed by fluid dynamics. This approach maintained the geometric and electrochemical stability of the biosensor during all stages of the detection process.

Impedance measurement parameters: Impedance spectra were recorded over a frequency range of 0.01 MHz to 10 MHz. Prior to measurements, the sensors were washed three times with PBS to remove unbound antibodies at the end of the antibody immobilization process. The first impedance recordings were then conducted using 150 μL of sterile human urine to establish the negative control baseline. After sample replacement with bacterial suspensions, further impedance measurements were performed at predetermined intervals (t = 15, 30, and 45 minutes). Throughout all measurements, the γ-bent geometry of the wire was preserved to maintain the stress-induced martensitic phase and the associated microcrack network.

1.6. Bacteria Detection

The ends of the gamma (ɣ)-bent NiTi sensor, which were left unmodified to serve as conductive contacts, were attached to the probes of EIS device for impedance measurement. A volume of 150 μL human urine was added to the sensor, and the first measurement was taken only after antibody binding. The system was allowed to stabilize for 45 minutes, during which negative control data were recorded.

The (ɣ)-bent NiTi surface, functionalized with thiol-based self-assembled monolayers (SAMs) and immobilized receptors, served as the electrochemical interface for impedance measurements. While the unmodified ends acted as conductive contacts, the central region exhibited localized electrochemical activity as a result of martensitic-phase microcracks formed under mechanical deformation.

These microcracks, partially filled with dielectric molecular coatings (MUA, EDC/Sulfo-NHS, and BSA), behaved collectively like a distributed network of nanoscale capacitive elements. Upon exposure to *E.coli*, specific binding between the bacterial cells and the immobilized receptors within these confined regions altered the dielectric environment. According to the parallel-plate capacitor model ($C = \varepsilon \frac{A}{d}$), such binding events can increase the local absolute permittivity (ε) or affect the effective gap distance (d), thereby modulating the overall capacitance (Figure S3). Here, ε represents the absolute permittivity of the local dielectric environment formed between the sensing surface and the bound analytes. Since impedance is inversely related to capacitance at certain frequencies, these microscopic changes accumulate and produce measurable shifts in the Nyquist plot — most notably in the semicircle diameter that reflects charge transfer resistance (Rct).

In addition, prior studies have demonstrated that bacterial adhesion to a functionalized metallic electrode surface directly impacts interfacial impedance [13]. When intact, bacterial membranes act as insulating barriers, impeding current flow and increasing impedance under low-frequency electric

fields. Meanwhile, at higher frequencies (in the MHz range), these fields can partially penetrate the membrane and interact with intracellular (cytoplasmic) conductivity [14]. This frequency-dependent behavior further enriches the impedance profile, enhancing the sensitivity and specificity of the detection mechanism.

1.7. Sensor Characterization

Dose response and sensitivity: To assess the dose-dependent behavior of the biosensor, functionalized NiTi wires were exposed to *E.coli* suspensions at six different concentrations (10^2, 10^3, 10^4, 10^5, 10^6, and 10^7 CFU/mL) in 150 μL volumes of fresh human urine. Impedance spectra were recorded at 15, 30, and 45 minutes after exposure. Post-incubation washing was intentionally omitted to preserve the structural integrity of the γ-bent sensor and prevent disturbance of the martensitic microcrack configuration. Since the sensor was mechanically confined, even minor perturbations caused by liquid exchange could potentially alter the configuration of stress-induced microcracks. Therefore, all detection data reflect the sensor response in the presence of unwashed bacterial samples, ensuring consistency in the measured impedance changes.

A total of 1003 raw data points were collected throughout the experiments. Only those shown in the figures were processed using a nonlinear parabolic curve fitting method. ΔRct values were calculated based on the intersection points of the fitted Nyquist plots with the real axis. All data analysis and fitting procedures were conducted using OriginPro (version 2019/b, OriginLab Corporation, USA). The resulting ΔRct (%) values exhibited a clear, concentration-dependent trend, highlighting the sensor's high sensitivity and consistent dose–response performance.

Selectivity: To determine the selectivity of the biosensor, additional impedance measurements were conducted using bacterial mixtures containing either only non-target species (Proteus mirabilis and Klebsiella pneumoniae, 10^5 CFU/mL each) or a combination of these species with *E.coli* (also at 10^5 CFU/mL). All samples were prepared in 150 μL of human urine. The sensors exhibited a pronounced increase in ΔRct only in the presence of *E.coli*, while the non-target group elicited minimal signal change. These results confirmed that the antibody-functionalized surface was highly selective for *E.coli*, with negligible cross-reactivity to other gram-negative bacteria tested under identical conditions.

Repeatability:

Table S1. Calibration Repeatability Results

Calibration repeatability results for the NiTi-based biosensor. Triplicate measurements were performed at each concentration. Mean values are reported with standard deviation (SD), standard error (SE), and relative standard deviation (%RSD).

Concentration (CFU/mL)	Sensor 1 ΔRct%	Sensor 2 ΔRct%	Sensor 3 ΔRct%	Mean ΔRct%	SD	SE	%RSD
10^2	68.98	56.87	65.97	63.94	6.31	3.64	9.87%
10^3	156.09	158.77	199.73	171.53	24.46	14.12	14.26%
10^4	422.18	396.55	384.33	401.02	19.32	11.15	4.82%
10^5	616.25	696.38	680.42	664.35	42.41	24.49	6.39%
10^6	811.56	720.99	715.4	749.32	53.98	31.16	7.20%
10^7	737.13	763.61	770.29	757.01	17.54	10.13	2.32%

References

1. Socrates, G., *Infrared and Raman characteristic group frequencies: tables and charts*. 2004: John Wiley & Sons.
2. Zhang, M., et al., *Sulfur-doping promoting peroxone reaction over TiO2 for highly effective NO oxidation at low temperature: Experimental and DFT studies.* 2022. **429**: p. 132475.
3. Love, J.C., et al., *Self-assembled monolayers of thiolates on metals as a form of nanotechnology.* 2005. **105**(4): p. 1103-1170.
4. Fontanesi, C., et al., *New one-step thiol functionalization procedure for Ni by self-assembled monolayers.* 2015. **31**(11): p. 3546-3552.
5. Loglio, F., et al., *Nickel sulfur thin films deposited by ECALE: Electrochemical, XPS and AFM characterization.* 2010. **638**(1): p. 15-20.
6. Sánchez-Bodón, J., et al., *Bioactive coatings on titanium: a review on hydroxylation, self-assembled monolayers (SAMs) and surface modification strategies.* 2021. **14**(1): p. 165.
7. Wang, J.-H. and M.J.E.C. Liu, *Computational study of sulfur–nickel interactions: A new S–Ni phase diagram.* 2007. **9**(9): p. 2212-2217.
8. Yeyin, T., et al., *Protection of nickel by self-assembled monolayers prepared in an aqueous self-emulsifying solution of a novel amphipathic organothiol.* 2023. **13**(7): p. 4331-4339.
9. Sun, T., et al., *Surface characteristics, properties and in vitro biological assessment of a NiTi shape memory alloy after high temperature heat treatment or surface H2O2-oxidation: a comparative study.* 2011. **130**(1-2): p. 45-58.
10. Tao, H., et al., *Effects of H2O2 pretreatment on surface characteristics and bioactivity of NaOH-treated NiTi shape memory alloy.* 2006. **16**(6): p. 1295-1300.
11. Soylu, M.C., et al., *Insulation by solution 3-mercaptopropyltrimethoxysilane (mps) coating: Effect of ph, water, and mps content.* 2013. **52**(7): p. 2590-2597.
12. Icoz, K., et al., *Quartz-crystal microbalance measurements of CD19 antibody immobilization on gold surface and capturing B lymphoblast cells: effect of surface functionalization.* 2018. **30**(5): p. 834-841.
13. Yang, L. and R.J.B.a. Bashir, *Electrical/electrochemical impedance for rapid detection of foodborne pathogenic bacteria.* 2008. **26**(2): p. 135-150.
14. Miura, T. and S.J.J.J.o.A.P. Uno, *Computer simulation for electrochemical impedance of a living cell adhered on the inter-digitated electrode sensors.* 2019. **58**(SB): p. SBBG15.
